# Health-related quality of life and QALY loss under COVID-19 lockdown: The case of Spain

**DOI:** 10.1371/journal.pone.0329413

**Published:** 2025-08-07

**Authors:** Mathieu F. Janssen, Kim Rand, Anabel Estévez-Carrillo, Juan Manuel Ramos-Goñi

**Affiliations:** 1 Maths In Health, Klimmen, The Netherlands; 2 Section Medical Psychology and Psychotherapy, Department of Psychiatry, Erasmus M.C., Rotterdam, The Netherlands; National Center for Global Health and Medicine, JAPAN

## Abstract

**Objectives:**

The COVID-19 pandemic forced many countries to implement confinement measures to limit the spread of the virus. Measuring the loss in terms of quality-adjusted life-years (QALYs) may provide a commensurable basis for comparing the impact of COVID-19. The aim of this research was to explore the impact of the first 21 days of COVID-19 lockdown on health-related quality of life (HRQoL) and associated QALY loss of the Spanish general population.

**Methods:**

A quota-based online survey was conducted in four waves with 500 general population respondents each: one conducted shortly before the lockdown (baseline) and 3 follow-ups conducted weekly. HRQoL data were collected using EQ-5D-5L. For comparison with pre-covid responses, data from the 2011–2012 National Health Survey was taken as reference. Data were analyzed using frequency analysis and logistic regression. QALY loss was estimated over the follow-up period and for the entire duration of the lockdown.

**Results:**

Comparing the baseline results to the follow-up results shows little change with respect to the distributions of reported problems in any of the 5 dimensions during the follow-up period. However, results for anxiety/depression show a 32% increase in the proportion of reported problems. The Spanish population was estimated to accrue a total of 1,994,216 QALYs over the study period. Based on the reference data, the population should have accrued 2,054,737 QALYs, leading to a loss of 60,520 QALYs over 21 days. For the entire lockdown, the corresponding loss would be 285,310 QALYs.

**Conclusions:**

A population under a lockdown situation reported higher rates of anxiety/depression problems than in a regular situation. On a country-wide scope, this may lead to a substantial loss in terms of QALYs, especially over longer periods of time. This is the first study to directly assess the impact of the lockdown in terms of QALY loss on a country-wide level.

## Introduction

On January 30^th^, 2020, the World Health Organization (WHO) declared COVID-19 a public health emergency of international concern, and elevated the threat level to “very high” on February 28^th^, 2020 [1]. Following the rapid spread of the virus, the WHO declared a global pandemic on March 11^th^, 2020 [2], and COVID-19 became an unprecedented challenge for national health and social welfare systems worldwide.

In Europe, the initial large-scale community transmission observed in Italy quickly spread to several other European countries, with high rates of early local transmissions observed in Austria, France, Germany, Switzerland and Spain in particular [3]. There were high hospital admission rates with a substantial number of patients needing ventilators and intensive care. As a result, national healthcare systems faced extreme demands to provide adequate care to infected persons. There was also the need for containment measures to limit risk of infection of health personnel and other patients. It was important to limit community transmission of the virus especially in high-risk groups such as the elderly in care facilities. Similar situations later occurred around the world [4]. Obviously, countries with less well-developed health-care infrastructure and weaker public institutions faced an increased risk of virus spread and subsequent impact on the population.

Within Europe, Spain quickly experienced an increased rate of hospitalizations threatening to outpace the capacity of the healthcare system, with fewer than 100 confirmed cases by the end of February 2020, rising to 6,391 confirmed cases and 196 deaths two weeks later. As a consequence, the Spanish government declared the State of Alarm (Royal Decree 463/2020) on March 14^th^ [5]. A battery of social, sanitary, and economic measures were put in place with the aim of reducing the impact during the sanitary emergency and trying to facilitate the recovery of socioeconomic indicators in a post-pandemic stage. The most salient of these measures was the nationwide lockdown imposed by the Spanish government on March 14^th^, 2020: home based confinement for the entire population, with exceptions made for health professionals, safety departments, and specific strategic manufacturers. Social exposure was kept at a minimum, with general permission to leave the home limited to the purchasing of basic goods such as food and medicine. Outdoor exercise or leisure activities in public spaces were strictly forbidden. Social distancing measures of various kinds, including lockdowns and travel restrictions, were implemented in most countries worldwide, with unprecedented consequences for commerce, tourism, and daily activities [4].

The implemented measures were deemed crucial to break chains of community transmission and avoid the collapse of hospitals through overload of available capacity in certain regions with a significant impact of the COVID-19 (i.e., Madrid, Catalunya, and Castilla-Leon). The measures were effective: the first wave reached a peak number of new confirmed cases (around 8,000 confirmed cases daily) between March 26^th^ and April 1^st^, and reached the peak number of deaths on April 2^nd^, at 961 people. However, as observed in many countries, the national lockdown had devastating immediate effects on the economy with a dramatic rise of observed and expected increase in unemployment rates and business bankruptcies [6]. The prolonged enforced confinement and the impact on welfare and economy came with a variety of adverse consequences. These consequences include a rise in the number of domestic abuse cases, and a well-documented increase in rates of mental health issues [7]. These issues come at the cost of a potential reduction in health-related quality of life (HRQoL), both in the short and longer term, as shown in previous infectious outbreaks (i.e., H1N1).

Measuring the impact of the pandemic and related policy measures on HRQoL is crucial for a variety of reasons: to fully understand the impact of the lockdown measures that have been implemented; to inform future public responses to health crises; and to build the knowledge base required to design successful policies for alleviating HRQoL loss following any future crisis. Measuring the loss in terms of quality-adjusted life-years (QALYs) may provide a commensurable basis for comparing the impact of COVID-19 and other threats to health and well-being.

The aim of this study was to explore the impact of the first 21 days of COVID-19-related confinement measures on the HRQoL of the Spanish general population. By way of example, we estimated QALY loss during the study period and additionally for the entire duration of the first lockdown. QALY impact was also assessed in terms of total accrued QALY loss from all COVID-19 related deaths until two weeks after the end of the study.

## Methods

### Study design and data collection

The first wave of data collection (i.e., the baseline data) was, by chance, conducted very shortly before the Spanish lockdown (12th March 2020). Three further waves of data collection were conducted, separated by weekly intervals.

Data were collected via an online survey. We conducted four consecutive waves using independent samples with one week’s space in between waves. Each wave included 500 adults of the Spanish general population, amounting to 2,000 respondents in total. Participants were recruited by a panel company and invited to participate in the study via a link to respond to the online questionnaire. A quota sampling approach was used, relying on a non-random selection of predetermined quota based on age, gender, and geographical location aimed to achieve representativeness of the Spanish general population. Each wave of sampling was independent, and any overlap of respondents between waves would be coincidental. Data collection period started on March 12^th^ and finished on April 14^th^, 2020. Participation was completely anonymous.

Ethical approval for the study was received within the umbrella of the EQ-5D-Y-3L valuation study in Spain project in Spain from Comité Ético de Investigación Clínica, Hospital Nuestra Señora del Prado, No. Dictamen 32/19. Written informed consent was obtained from all individual participants included in the study.

For comparison with pre-COVID-19 responses, we used data collected alongside the Spanish national health survey in 2011–2012 as reference. These data are freely accessible from the Spanish national institute of statistic (INE) [8].

### Outcome measures

We used the EQ-5D-5L, which is the most used instrument worldwide for measuring HRQoL for use in health economic assessment [9,10], and the EQ-5D-Y-5L. Due to its brevity, well-documented measurement properties in a variety of populations and areas (including the general population and mental health populations) [11] and international uptake, the EQ-5D is particularly suited for our study aims, further strengthened by a recent systematic review on the use of EQ-5D-5L in assessing the impact of post-acute COVID-19 syndrome [12]. The EQ-5D-5L consists of five one-item dimensions: mobility, self-care, usual activities, pain/discomfort, and anxiety/depression, with dimensions describing levels of problems as ‘no’, ‘slight’, ‘moderate’, ‘severe’, and ‘unable to/extreme’ problems. The EQ VAS, an integral part of EQ-5D, measures health on a rating scale anchored at 0 (the worst health you can imagine) and 100 (the best health you can imagine). The EQ-5D asks respondents about their health on the day of completion. Value sets, providing values (utilities) for each health profile (any combination of responses to the five items), are available for many countries, reflecting the societal preferences for the corresponding country. Utility values based on the EQ-5D-5L descriptive system responses were calculated based on the Spanish EQ-5D-5L value set [13].

The EQ-5D-5L was used for the second, third and fourth wave of the study, along with demographic characteristics (age, gender, and geographical location). The first wave was conducted as part of the EQ-5D-Y-3L valuation study in Spain [14]. In this study, a representative sample of the Spanish adult population was used, and data collection included not only the EQ-5D-Y-3L but also the EQ-5D-Y-5L instrument. The EQ-5D-Y-5L is a version of EQ-5D suitable for children and adolescents, but an adult sample was used in this study for valuation purposes. The EQ-5D-Y-5L covers the same five dimensions as the EQ-5D-5L, although several dimension headers were adapted, such as including ‘going to school’ for usual activities, and using ‘feeling worried, sad or unhappy’ instead of ‘anxiety/depression’. The response labels use more child-friendly terms for levels 3 and 4 (‘’some’/’quite’ and ‘a lot of’/’really’). Given the similarities between the EQ-5D-5L and EQ-5D-Y-5L we considered these data as proxy for EQ-5D-5L data. Furthermore, the EQ-5D-5L instrument was used in the National Health Survey of Spain (2011/2012) [8], therefore, pre-COVID-19 population norms conveniently detailed were available for the Spanish population [15]. These data were used as reference data for the current study.

We used dimensions scores (level responses and mean level scores, calculated as the mean of the level responses by dimension with no problems coded as 1, slight problems as 2, etc), EQ VAS and utility values as outcome measures for this study.

### Analysis

The study samples were described according to gender, age, and geographical location using absolute and relative frequencies. We classified geographical location by their severity of COVID-19 incidence into 3 groups (mild, moderate, severe). This classification of regional epidemic severity was defined by tertile subgroups based on the rate of cases per 100,000 members of the population by geographical location. Rates lower than 201 cases were classified as mild, rates between 201 and 544 cases were classified as moderate and rates over 544 cases were classified as severe. Additionally, age groups were also defined (18–29, 30–59 and ≥60 years). These two variables defined the sample subgroup used to provide a concise and easy-to-read overview. Gender, age and regional epidemic severity (3 groups) for the pooled data were statistically compared with the reference data using the chi-square test for nominal variables (gender and regional epidemic severity) and the Mann-Whitney *U* test for ordinal variables (age groups). We hypothesized that HRQoL would be lower for higher age groups and for regions with increased epidemic severity.

EQ-5D-5L responses were described using the percentage of patients reporting each level of functioning on each dimension over the follow-up period. Furthermore, to evaluate the association of the sociodemographic variables with HRQoL, results were also calculated for the subgroups defined above. Differences in reported levels of problems by EQ-5D-5L dimension between the total lockdown sample and the general population sample from the reference data were compared using the independent samples t-test and weighted proportional odds logistic regression. A main effects model was used, estimating the log-odds of reporting more problems on each EQ-5D dimension for respondents reporting during lockdown versus no lockdown (i.e., year of data collection) and age group. A second model additionally included interaction terms for lockdown and age. We hypothesized that HRQoL would be lower (i.e., more reported problems and lower EQ VAS scores) during the lockdown period when compared to the reference period, specifically for usual activities, pain/discomfort and anxiety/depression, resulting in overall lower utilities. No hypotheses were formulated for how HRQoL would develop during the lockdown period, as the period was relatively short and the effect of a lockdown of this scale was hard to predict because of its novelty.

Boxplots were used for graphical representation of health differences in utility values and VAS scores. Responses from the four waves were combined and compared with the reference data.

By way of example, we estimated QALY-loss on the study data collection period (3 weeks). QALY-loss was estimated by taking the difference between utility values from the reference data (the 2011/2012 NHS study) and utility values from the four surveys, both stratified by age group (18−29 years, 30−59 years, 60 + years), sex, and regional COVID-19 rate severity. Resulting utility differences were applied to the adult Spanish 2020 adult population (N = 39,006,106) [16] to arrive at the estimated QALY loss for the duration of the study by multiplying utility differences with the study duration. We also estimated the QALY-loss for the entire duration of the lockdown (March 14^th^ until June 21^st^, 16) by taking the mean QALY-loss for the 4 waves and extrapolating to the duration of the lockdown, assuming utilities would remain the same on average. For a useful reference showing how QALY losses can be calculated as a result of COVID-19, see Briggs et al (2021) [17].

Additionally, we estimated the total QALY-loss associated with premature deaths due to COVID-19 in Spain up until April 28th (two weeks after the final survey round), as reported by the Spanish Ministry of Health, Consumption, and Social Welfare (MSCBS) [18,19]. In this comparison, we assumed that all individuals reported as having deceased due to COVID-19 would otherwise have had a remaining life expectancy equal to that of the adult general population, stratified by sex and yearly age group (Spanish institute of statistics (INE), table 27153 [20]). For each expected remaining year of life, they were assumed to accrue QALYs based on the utility mean reported in the baseline study data, stratified by sex and age group (18–29 years, 30–59 years, 60 + years). As the QALY deaths reported by MSCBS were for 10-year age brackets, expected QALYs per sex/age bracket were calculated as the mean weighted by the size of the adult Spanish population in each sex/yearly age group, as reported in INE table 31304 [16].

## Results

Demographic characteristics for the participants in the four waves can be found in Table 1, juxtaposed with corresponding numbers from the reference data (2011–2012 NHS study). The pooled study sample was younger than the reference data based on the general population and contains more males (51%) compared to the reference sample (46%). Differences between the samples were statistically significant for both gender and age (P < 0.001), with the largest differences observed for the oldest age groups. The geographical spread showed similar results between the study sample and the reference sample and were not significantly different.

**Table 1:** Respondent characteristics by sampling time, and Spanish population >=18 years.

		Lockdown study										Reference data		Spain adult population 2020	
		Baseline		+1 week		+2 weeks		+3 weeks		Pooled		NHS 2012			
**Total**		500	(100%)	509	(100%)	500	(100%)	501	(100%)	2,010	(100%)	20,560	(100%)	38,944,956	(100%)
**Gender**															
Male		275	(55%)	257	(50.5%)	256	(51.2%)	246	(49.1%)	1,034	(51.4%)	9,397	(45.7%)	18,875,906	(48.5%)
Female		225	(45%)	252	(49.5%)	244	(48.8%)	255	(50.9%)	976	(48.6%)	11,163	(54.3%)	20,069,050	(51.5%)
**Age group**															
18–24 years		48	(9.6%)	36	(7.1%)	36	(7.2%)	36	(7.2%)	156	(7.8%)	1,235	(6%)	948,848	(2.4%)
25–29 years		70	(14%)	42	(8.3%)	42	(8.4%)	37	(7.4%)	191	(9.5%)	1,125	(5.5%)	4,885,287	(12.5%)
30–39 years		128	(25.6%)	125	(24.6%)	131	(26.2%)	123	(24.6%)	507	(25.2%)	3,640	(17.7%)	6,091,315	(15.6%)
40–49 years		113	(22.6%)	127	(25%)	134	(26.8%)	131	(26.1%)	505	(25.1%)	3,822	(18.6%)	7,822,003	(20.1%)
50–59 years		69	(13.8%)	104	(20.4%)	104	(20.8%)	110	(22%)	387	(19.3%)	3,311	(16.1%)	7,023,995	(18%)
60–69 years		53	(10.6%)	65	(12.8%)	44	(8.8%)	54	(10.8%)	216	(10.7%)	3,033	(14.8%)	5,343,036	(13.7%)
70 + years		19	(3.8%)	10	(2%)	9	(1.8%)	10	(2%)	48	(2.4%)	4,394	(21.4%)	6,830,472	(17.5%)
**Autonomous community**	*Epidemic severity*														
Andalucia	Mild	73	(14.6%)	87	(17.1%)	87	(17.4%)	87	(17.4%)	334	(16.6%)	2,439	(11.9%)	6,875,991	(17.7%)
Aragon	Moderate	19	(3.8%)	20	(3.9%)	20	(4%)	20	(4%)	79	(3.9%)	846	(4.1%)	1,106,129	(2.8%)
Asturias	Moderate	23	(4.6%)	18	(3.5%)	20	(4%)	20	(4%)	81	(4%)	814	(4%)	884,840	(2.3%)
Baleares	Mild	6	(1.2%)	10	(2%)	10	(2%)	10	(2%)	36	(1.8%)	710	(3.5%)	997,798	(2.6%)
Canarias	Mild	17	(3.4%)	23	(4.5%)	23	(4.6%)	23	(4.6%)	86	(4.3%)	1,035	(5%)	1,881,512	(4.8%)
Cantabria	Moderate	4	(0.8%)	10	(2%)	10	(2%)	10	(2%)	34	(1.7%)	742	(3.6%)	490,015	(1.3%)
Castilla Leon	Severe	32	(6.4%)	41	(8.1%)	32	(6.4%)	32	(6.4%)	137	(6.8%)	1,274	(6.2%)	2,055,215	(5.3%)
Castilla Mancha	Severe	16	(3.2%)	27	(5.3%)	20	(4%)	20	(4%)	83	(4.1%)	1,011	(4.9%)	1,675,335	(4.3%)
Cataluña	Severe	69	(13.8%)	51	(10%)	51	(10.2%)	51	(10.2%)	222	(11%)	2,233	(10.9%)	6,240,689	(16%)
Comunidad Valenciana	Moderate	47	(9.4%)	37	(7.3%)	38	(7.6%)	38	(7.6%)	160	(8%)	1,667	(8.1%)	4,135,882	(10.6%)
Extremadura	Moderate	6	(1.2%)	18	(3.5%)	18	(3.6%)	18	(3.6%)	60	(3%)	843	(4.1%)	886,861	(2.3%)
Galicia	Moderate	44	(8.8%)	45	(8.8%)	45	(9%)	45	(9%)	179	(8.9%)	1,234	(6%)	2,318,203	(6%)
Madrid	Severe	92	(18.4%)	57	(11.2%)	59	(11.8%)	59	(11.8%)	267	(13.3%)	1,890	(9.2%)	5,515,904	(14.2%)
Murcia	Mild	21	(4.2%)	25	(4.9%)	25	(5%)	26	(5.2%)	97	(4.8%)	779	(3.8%)	1,197,813	(3.1%)
Navarra	Severe	5	(1%)	5	(1%)	5	(1%)	5	(1%)	20	(1%)	755	(3.7%)	534,778	(1.4%)
Pais Vasco	Severe	19	(3.8%)	31	(6.1%)	33	(6.6%)	33	(6.6%)	116	(5.8%)	1,168	(5.7%)	1,822,965	(4.7%)
La Rioja	Severe	6	(1.2%)	3	(0.6%)	3	(0.6%)	3	(0.6%)	15	(0.7%)	690	(3.4%)	261,056	(0.7%)
Ceuta Melilla	Mild	1	(0.2%)	1	(0.2%)	1	(0.2%)	1	(0.2%)	4	(0.2%)	430	(2.1%)	63,970	(0.2%)

NHS National Health Survey.

Epidemic severity was based on tertile subgroups of rates of cases per 100,000 members of the population by geographical location

Comparing the study results to those from the reference data shows that for mobility the proportion of respondents reporting no problems with mobility are lower for the study sample (13.9%) than for the reference sample (17.8%), and that the respondents that do report problems in the study sample tend to report less severe problems (Table 2). Differences were statistically significant (P < 0.001). Mean (SD) level scores for mobility were 1.19 (0.54) for the study sample and 1.32 (0.79) for the reference sample. The results for self-care and usual activities were similar between both samples and not statistically significant. For pain/discomfort more reported problems were observed by the study sample (37.0%) compared to the reference sample (28.7%), although mean level scores were similar (both 1.48). However, results for anxiety/depression show a 32% increase in the proportion of reported problems with 47.6% reported problems in the study samples versus 16.6% for the reference sample. An increase is reported at all severity levels, although most prominently for level 2 (slightly anxious or depressed) from 9% to 35%. The mean level score increased to 1.67 compared to 1.27 for the reference sample. Differences between study and reference samples were significant for pain/discomfort and anxiety/depression (P < 0.001).

**Table 2:** Number of participants, problems by EQ-5D-5L dimension, level, and data collection period.

	2020					2012
	BL	FU1	FU2	FU3	Total	
*N*	500	509	500	501	2,010	20,560
	**2020**					**2012**
**Mobility**	**BL**	**FU1**	**FU2**	**FU3**	**Total**	
No problems	87.4%	87.4%	86.6%	82.8%	86.1%	82.2%
Slight problems	9.4%	10.4%	8.8%	12.0%	10.1%	8.0%
Moderate problems	2.0%	1.8%	3.6%	3.6%	2.7%	5.8%
Severe problems	0.8%	0.4%	0.8%	1.2%	0.8%	3.2%
Extreme problems	0.4%	0.0%	0.2%	0.4%	0.2%	0.8%
**Self-care**						
No problems	92.8%	93.3%	93.0%	90.2%	92.3%	92.0%
Slight problems	5.2%	6.3%	4.4%	6.2%	5.5%	3.6%
Moderate problems	1.8%	0.4%	1.8%	3.0%	1.7%	2.2%
Severe problems	0.0%	0.0%	0.6%	0.6%	0.3%	1.2%
Extreme problems	0.2%	0.0%	0.4%	0.0%	0.1%	1.0%
**Usual Activities**						
No problems	85.4%	84.1%	85.8%	81.8%	84.3%	86.2%
Slight problems	10.4%	12.2%	8.6%	13.2%	11.1%	6.4%
Moderate problems	3.0%	2.8%	3.4%	3.4%	3.1%	4.0%
Severe problems	1.0%	0.2%	0.8%	1.4%	0.8%	1.9%
Extreme problems	0.2%	0.8%	1.4%	0.2%	0.6%	1.6%
**Pain/discomfort**						
No problems	61.6%	60.1%	62.2%	68.1%	63.0%	71.3%
Slight problems	28.4%	30.8%	27.4%	23.6%	27.6%	14.3%
Moderate problems	7.8%	7.9%	8.2%	7.2%	7.8%	10.0%
Severe problems	2.2%	0.8%	1.8%	1.2%	1.5%	4.0%
Extreme problems	0.0%	0.4%	0.4%	0.0%	0.2%	0.5%
**Anxiety/depression**						
No problems	51.6%	51.1%	55.6%	51.3%	52.4%	83.4%
Slight problems	34.0%	32.8%	31.6%	34.7%	33.3%	9.4%
Moderate problems	10.0%	11.4%	9.0%	9.4%	10.0%	4.8%
Severe problems	3.8%	3.7%	2.8%	3.4%	3.4%	1.9%
Extreme problems	0.6%	1.0%	1.0%	1.2%	0.9%	0.5%

BL baseline, FU follow-up.

**Fig 1:**
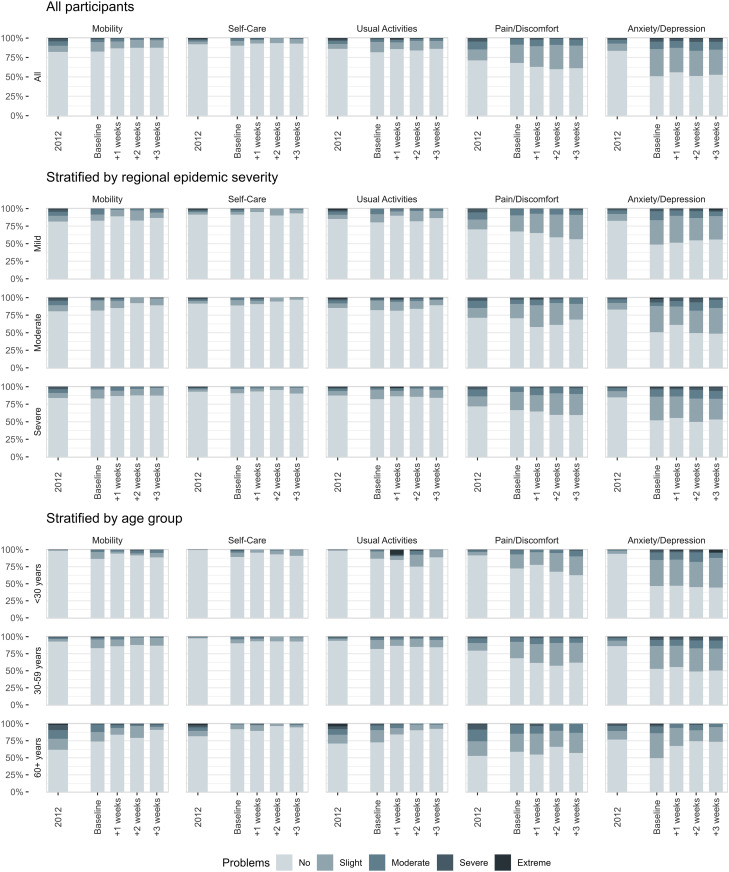
Proportion of respondents reporting each level of health by EQ-5D dimension (total and stratified by regional epidemic severity and age). Regional epidemic severity was defined by tertile subgroups of rates of cases per 100,000 members of the population by geographical location.

The distributions of reported problems in any of the 5 dimensions were similar when comparing the baseline results to the follow-up results (Fig 1). For the pooled data, differences in dimension scores between regional epidemic severity were not statistically significant. For the 3 main age groups significant differences were found for mobility, pain/discomfort and anxiety/depression for the pooled data, with more reported problems with increasing age for mobility and pain/discomfort while for anxiety/depression the pattern was reversed, with a mean (SD) level score for the youngest group of 1.73 (0.84), while for the oldest age group a mean level score of 1.45 (0.71) was observed.

**Fig 2:**
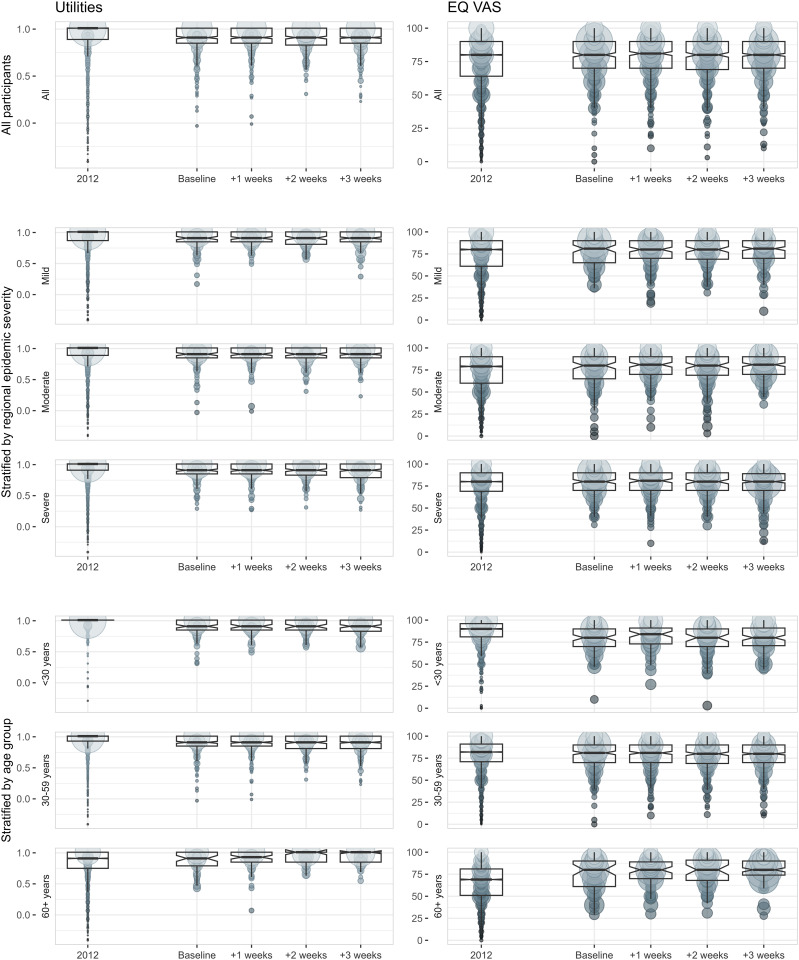
Distribution of utility and EQ VAS by time, regional epidemic severity, and age represented by notched box-plots*. Regional epidemic severity was defined by tertile subgroups of rates of cases per 100,000 members of the population by geographical location. * Boxes represent inter-quartile ranges while notches indicate 95% confidence interval around the median. Colored circle areas represent the proportion of respondents assigning a particular value.

A mean (SD) EQ VAS score of 78.0 (16.2) was observed for the pooled study sample, significantly higher (P < 0.001) when compared to the reference sample with a score of 75.4 (18.9). Mean (SD) utility values were 0.89 (0.14) for the pooled study sample and 0.91 (0.18) for the reference sample, with a statistically significant difference (P < 0.001). When comparing baseline results to the 3 follow-up results, neither EQ VAS scores nor utility values were significantly different between any time point (Fig 2). For the pooled data, there were no significant differences in EQ VAS scores and utility values between the 3 main epidemic severity regions or the 3 main age groups.

Two sets of ordered logistic regression models were used to model the extent to which respondents in 2020 differed from 2012 in terms of levels of problems reported for each EQ-5D dimension (Table 3). In the first model, the predictors included were responding in 2020, and age groups 30–59 and 60 + . In the second, interaction between 2020 and age groups were included. The models indicate a strong tendency for higher age respondents to report more problems on all EQ-5D dimensions. The model including age groups and no interaction terms further indicates that respondents in 2020 had significantly higher odds of reporting problems on usual activities, pain/discomfort, and anxiety depression. Including year 2020 x age group interaction reveals that this tendency was significantly greater in the youngest age group, which reported statistically significantly more problems on all EQ-5D dimensions.

**Table 3:** Weighted proportional odds logistic regression comparing EQ-5D-5L dimension levels for 2012 and 2020. Reference group: age 18-29, no lockdown.

Model 1: Main effect of lockdown and age										
	Mobility		Self-care		Usual Activities		Pain/discomfort		Anxiety/depression	
	Estimate (SE)	*p*	Estimate (SE)	*p*	Estimate (SE)	*p*	Estimate (SE)	*p*	Estimate (SE)	*p*
Lockdown	−0.146 (0.07)	0.037	0.082 (0.091)	0.372	0.288 (0.067)	<0.001	0.432 (0.048)	<0.001	1.406 (0.047)	<0.001
Age 30–59	1.233 (0.117)	<0.001	0.835 (0.164)	<0.001	0.903 (0.109)	<0.001	0.898 (0.061)	<0.001	0.589 (0.062)	<0.001
Age 60+	3.119 (0.115)	<0.001	2.774 (0.157)	<0.001	2.542 (0.106)	<0.001	2.02 (0.061)	<0.001	1.045 (0.063)	<0.001
**Model 2:** Including interaction between lockdown and age group										
	Mobility		Self-care		Usual Activities		Pain/discomfort		Anxiety/depression	
	Estimate (SE)	*p*	Estimate (SE)	*p*	Estimate (SE)	*p*	Estimate (SE)	*p*	Estimate (SE)	*p*
Lockdown	1.778 (0.241)	<0.001	2.559 (0.314)	<0.001	2.515 (0.212)	<0.001	1.404 (0.139)	<0.001	2.632 (0.127)	<0.001
Age 30–59	1.493 (0.144)	<0.001	1.348 (0.236)	<0.001	1.41 (0.152)	<0.001	1.026 (0.069)	<0.001	0.894 (0.079)	<0.001
Age 60+	3.539 (0.141)	<0.001	3.541 (0.23)	<0.001	3.235 (0.149)	<0.001	2.251 (0.069)	<0.001	1.501 (0.08)	<0.001
Year 2020 * Age 30–59	−1.078 (0.259)	<0.001	−1.344 (0.339)	<0.001	−1.523 (0.232)	<0.001	−0.672 (0.153)	<0.001	−1.021 (0.142)	<0.001
Year 2020 * Age 60+	−2.778 (0.263)	<0.001	−3.575 (0.35)	<0.001	−3.193 (0.238)	<0.001	−1.614 (0.16)	<0.001	−2.025 (0.152)	<0.001

SE standard error.

**Fig 3:**
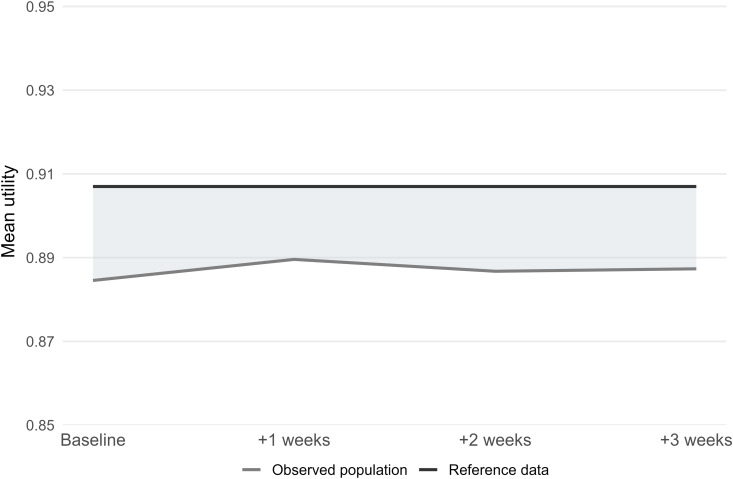
QALYs lost representation due to COVID-19 lockdown in Spain over the three week study period. BL baseline, FU follow-up, QALY quality-adjusted life year.

Over the 3 weeks covered by the four study waves, the Spanish population was estimated to accrue a total of 1,994,216 QALYs (Fig 3 and Table 4). Based on corresponding HRQoL data from 2011/2012, the population should have accrued a total of 2,054,737 QALYs. The difference indicates a loss for the general Spanish population of 60,520 QALYs over a 3-week period. As the situation observed in these first weeks of the Spanish lockdown prolonged for 99 days, the corresponding loss would be 285,310 QALYs.

**Table 4:** QALYs lost estimation due to the COVID-19 lockdown in Spain.

Sex	Age group	Regional epidemic severity	2020 Spanish adult population	Mean utility reference data	Mean utility study data	Expected QALYs (reference data)	Estimated QALYs (study data)	QALY difference
Male	18-29 years	Mild	925,957	0.98	0.91	908,926	845,747	63,179
Female	18-29 years	Mild	881,678	0.97	0.90	859,180	795,525	63,656
Male	30-59 years	Mild	3,100,452	0.95	0.90	2,947,774	2,796,001	151,774
Female	30-59 years	Mild	3,069,644	0.93	0.89	2,845,264	2,728,871	116,394
Male	60 + years	Mild	1,410,298	0.87	0.87	1,223,153	1,232,467	−9,314
Female	60 + years	Mild	1,690,205	0.75	0.82	1,265,620	1,389,265	−123,645
Male	18-29 years	Moderate	682,794	0.98	0.86	668,584	586,026	82,558
Female	18-29 years	Moderate	649,714	0.98	0.91	639,095	591,613	47,482
Male	30-59 years	Moderate	2,571,574	0.96	0.91	2,460,440	2,332,172	128,268
Female	30-59 years	Moderate	2,560,455	0.94	0.86	2,398,442	2,192,198	206,244
Male	60 + years	Moderate	1,498,699	0.88	0.89	1,320,807	1,338,676	−17,869
Female	60 + years	Moderate	1,858,694	0.79	0.93	1,462,045	1,721,451	−259,405
Male	18-29 years	Severe	1,375,444	0.98	0.89	1,351,921	1,227,546	124,375
Female	18-29 years	Severe	1,332,681	0.98	0.88	1,303,434	1,170,517	132,917
Male	30-59 years	Severe	4,805,803	0.96	0.89	4,635,966	4,297,274	338,692
Female	30-59 years	Severe	4,862,419	0.94	0.87	4,555,843	4,219,205	336,637
Male	60 + years	Severe	2,535,240	0.90	0.91	2,280,852	2,305,838	−24,985
Female	60 + years	Severe	3,194,355	0.81	0.91	2,585,934	2,890,990	−305,057
						**QALYs**		**QALYs lost**
**Total population**			39,006,106		Study duration	2,054,737	1,994,216	60,520
					Lockdown duration	9,686,616	9,401,306	285,310

QALY quality-adjusted life year.

Additionally, the estimated QALY-loss associated with the 15,956 COVID-19-related deaths in Spain until April 28^th^ 2020, assuming that they would otherwise have lived to the estimated life expectancy for their corresponding age/sex group, with HRQoL corresponding to their respective mean based on the NHS study, would tally 146,584.

## Discussion

This study assesses the HRQoL impact on the general population of a country-based lockdown under a pandemic situation. Specifically, we present EQ-5D-5L outcomes of the Spanish general population during the first month of the COVID-19 pandemic lockdown occurred in Spain. To our knowledge this is the first study to assess the impact of the lockdown in terms of QALY loss on a country-wide level. Since many countries adopted comparable measures against COVID-19 compared to Spain, our findings could be extrapolated to other jurisdictions which have adopted similar rules.

EQ-5D-5L results over the four points in time in the early lockdown did not show much variation in terms of dimension scores, utilities and EQ VAS scores. This might have been expected for various reasons. Since the time periods between intervals was so short (1 week), any effect on functional dimension (including pain) is expected to be minimal. In terms of impact on mental health (anxiety/depression), the general population was already informed the lockdown would be in place so most people probably mentally prepared for this, anticipating isolation for an unknown period of time. Subgroup analyses showed that although there were no differences in three main age groups in terms of utilities for the pooled data, there were significantly more reported problems in mobility and pain/discomfort, but less reported problems in anxiety/depression with increasing age, as hypothesized. Apparently, these effects were cancelled out when combining the dimension scores in utilities. Surprisingly, regions with increased epidemic severity showed no additional impact on HRQoL, contrary to our hypotheses, which may indicate that the effect of the lockdown (e.g., the isolation) was larger than the impact of increased COVID-19 incidence rates in the region where participants resided.

Differences in the study results with the 2012 reference data were larger, with notably more reported problems for anxiety/depression during lockdown, as hypothesized, which can be explained because of the effect of the lockdown in particular and the pandemic in general, which was also observed elsewhere [21–25]. Note that it would be challenging to try to disentangle these two related effects. There also were more reported problems in pain/discomfort during lockdown, also in accordance with our hypotheses, although the mean level score was identical as less severe problems were reported compared to the reference data, while for mobility there were less reported problems during the lockdown. As a result, utilities were also slightly lower during lockdown when compared to the reference sample. Interestingly, and contrary to our hypotheses, an opposite result was observed for EQ VAS scores that were higher during lockdown. Although this might seem inconsistent, the EQ VAS is likely to capture a wider or different concept of HRQoL compared to the dimensions of EQ-5D-5L, which is also demonstrated by previously reported relatively low correlations between EQ VAS and utilities [26]. Furthermore, although statistically significant, the mean difference in EQ VAS scores between the lockdown sample and the reference sample was only 2.6, which is smaller than commonly reported minimal important differences [27]. No increased number of reported problems were observed for usual activities during the lockdown period. Apparently participants did not associate problems with usual activities with the restrictions imposed by the lockdown, perhaps by focusing on their capabilities to perform usual activities, that were not affected by the lockdown. Overall, results were comparable to results from other studies conducted during the pandemic. In a multi-country general population study by König et al (2023) [25], EQ-5D-5L utilities were observed ranging from 0.80 (Denmark) to 0.94 (France) and EQ VAS scores ranging from 70.1 (United Kingdom) to 78.4 (Portugal). Note that differences in utilities between countries are also caused by differences between value set characteristics for different countries. Ferreira et al (2021) conducted a similar study to ours, comparing the Portuguese general population under lockdown with a pre-pandemic sample from 2015/2016 [28]. Results were strikingly similar, with an EQ-5D-5L mean utility of 0.861 during lockdown and 0.887 for the pre-pandemic reference sample, resulting in an overall utility difference of 0.026, similar to the mean utility difference we observed (0.022). Again, note that absolute differences between utilities may result from differences between the Portuguese and Spanish value sets.

The exploratory QALY calculations in the current study were intended as an example, showing that even with a relatively small difference in utilities the total amount of QALYs will amount to substantial proportions, as a result of the impact of the pandemic and the lockdown affecting entire populations of a particular country. Differences in utilities between the lockdown sample and the reference sample might have been relatively small because the reference data were collected during a period of economic crisis, which also may have impacted HRQoL. The QALY loss in the general population observed in this study is likely attributable to a number of different factors, including direct and indirect effects of quarantine-measures (e.g., isolation, lack of freedom), fear of COVID-19, indirect suffering due to health problems of others, and anxiety related to economic downturn. This study alone is not suited to determine the extent to which these and other factors are at play. What factors are at play is far from trivial. For instance, if the QALY-loss is driven by quarantine-related effects and consequent economic downturn, countries with similar lockdowns could see corresponding QALY losses while such measures are in place, irrespective of whether the infection-reducing efforts were successful. If, however, the primary drivers are related to economic impacts, there may be substantial differences in loss of HRQoL between countries as a function of what political and economic countermeasures were implemented to prevent or ameliorate economic impact on the population.

The estimated QALY-loss, adding up to approximately 60,000 QALYs over just 3 weeks, was considerable. Considering that participation in the study likely indicated relatively good health, this QALY-loss presumably reflects HRQoL loss suffered by those not directly afflicted by COVID-19. In addition to this and the QALY loss from premature deaths, there is a likely much larger QALY-loss associated with both short and long-term health problems accrued by COVID-19 survivors. A study calculating QALY loss due to COVID-19 related deaths in Spain led to similar results, ranging between 90,762 and 184,359 QALYs lost (based on 28,000 deaths) [29]. It must also be noted that there are several known criticisms and challenges with the QALY concept and its application in general, including methodological issues (e.g., whose values to use, whether to include nonhealthy effects), the neutral application of QALYs (e.g., equity considerations, issues with the aggregation of outcomes and individuals) and the risk of discrimination (e.g., against the elderly) [30].

It should be noted that the reference sample was dating back to 2012, Spain was suffering from an economic crisis. The difference in the 2012 utilities and those of the current study were relatively small, but could have expected to be larger if population reference data would have been available from a period outside of any (economic) crisis. In this sense, the estimates in the current study can considered to be conservative. Assuming all things equal between the two periods of data collection, it could be argued that the lockdown situation has had a greater impact on HRQoL than the economic crisis.

There are a few limitations to acknowledge in the current study. Firstly, differences of the current study results with the reference sample might be due to other factors than the lockdown or the pandemic. The NHS reference data used a different mode of administration and was based on a much larger sample. Moreover, the reference sample dates back to 2012, and there may be multiple reasons why HRQoL may have changed over this time period, such as cohort effects or demographic changes in the population. A second limitation is the relatively small sample size of the data collection under lockdown, which may have played a role in results being non-significant in some of the comparisons. Thirdly, an online panel company was used for data collection, which may have led to selection bias, as panel members tend to have higher educational attainment and socioeconomic status compared to non-panel members [31]. Fourth, the EQ-5D-Y-5L was used as proxy for the EQ-5D-5L for the baseline samples, which may have caused a bias in the results. However, since differences between baseline and follow-up results were minimal, we assume any bias will also have been minimal. Fifth, as mentioned above, the follow-up period for the current study was rather short, spanning a period of only 3 weeks. A large impact of the lockdown may have been measured with longer follow-up periods. Sixth, although the quota sampling method may have biased representativeness as it is based on convenience sampling rather than random (probability) sampling, a recent study comparing probability versus nonprobability (e.g., quota) sampling methods found comparable estimates of physical and mental health outcomes [32], implying the bias would be minimal. Seventh, mean level scores were used for EQ-5D-5L dimensions for part of the analysis, assuming parametric properties for an ordinal response scale. Finally, while the main aim of the study was to assess the impact of the lockdown, the baseline data were collected shortly before the lockdown. For that reason it could be argued that for studying solely the effects of the confinement measures, the baseline data could have been used as reference. However, it is plausible that people were anticipating the lockdown and already stayed home as much as possible before the measures were effective. Moreover, there already likely was a psychological effect of being confined present due to this anticipation.

To our knowledge this is the first study to assess the impact of the COVID-19 lockdown in terms of QALY loss on a country-wide level. There have been studies estimating QALY loss for specific conditions, using different methods to calculate disutilities [33–35]. Our approach, using population norm reference data, is novel.

## Conclusions

A population under lockdown reported higher rates of problems with anxiety/depression when compared to a normal situation. On a country-wide scope, this may lead to a substantial loss in terms of QALYs, especially over longer periods of time. The negative impacts of lockdowns are now quite well documented, and should be emphasised in any future discussions of similar potential interventions.
